# A Very Rare Presentation of Multiple Myeloma: Unilateral Raccoon Eye

**DOI:** 10.3889/oamjms.2015.073

**Published:** 2015-08-19

**Authors:** Ceyhun Varım, Hasan Ergenc, Mehmet Sevki Uyanık, Tezcan Kaya, Ahmet Nalbant, Cengiz Karacaer, Cenk Sunu, Ali Tamer

**Affiliations:** 1 *Sakarya University, Medical Faculty, Internal Medicine, 54000 Sakarya, Turkey*; 2 *Sakarya University Education and Research Hospital, Internal Medicine, 54000 Sakarya, Turkey*; 3 *Sakarya University Education and Research Hospital, Internal Medicine, Hematology, 54000 Sakarya, Turkey*

**Keywords:** raccoon eye, erythrocyte sedimentation rate, multiple myeloma, ecchymotic lesion, hematologic malignancies

## Abstract

Multiple myeloma (MM), the second most common hematological malignancy, is caused by the accumulation of monoclonal plasma cells in bone marrow. It accounts for 10–15% of deaths from hematological malignancies and approximately 2% of deaths from cancer. The median age at presentation is 70 years old. The diagnosis is incidental in 30% of cases. MM is often discovered through routine blood screening with a large gap between the total protein and the albumin levels.

Two thirds of patients complain of bone pain, especially lower back pain. MM could be diagnosed after a pathologic fracture occurs in one third of patients. Presentation with symptoms related to hyperviscosity, hypercalcemia and bleeding tendency could also be observed. A rare presentation of MM is peri-orbital ecchymotic lesion (raccoon eye). Here, we report a 64 years old, male patient presented with unilateral raccoon eye and high erythrocyte sedimentation rate (ESR) to internal medicine outpatient. The patient was referred to hematology outpatient and was diagnosed with multiple myeloma.

## Introduction

Multiple Myeloma (MM) is the second most observed hematologic malignancy due to accumulation of monoclonal plasma cells originating from the bone marrow in adults [1]. MM constitutes 10% of all hematologic malignancies. MM, solitary extramedullary plasmacytoma and bone plasmacytoma are the subgroups of plasma cell neoplasms [2]. Patients are usually asymptomatic in early stages, and bone pain is the main complaint within symptomic MM patients. Most commonly observed laboratory abnormalities are composed of anemia, hypercalcemia, hyperuricemia, proteinuria, nephrotic syndrome and acute and/or chronic renal failure. Hepatomegaly, splenomegaly or lymphadenopathy are observed only about 1-2 % of patients in the physical examination [3].

The triad of unilateral or bilateral rapidly progressive proptosis, periorbital ecchymosis and edema are called raccoon eyes [4]. This typical presentation could be seen in the skull base fracture, orbital metastasis of solid malignancies, myxedema, amyloidosis, multiple myeloma, lymphoma and hemophilia. Infiltration of amyloid protein in the capillary, and capillary fragility are thought to be the pathophysiological mechanisms underlying raccoon eyes in MM. So that these capillaries can burst, even after minor stress, resulting in raccoon eyes [5].

Here, we present a case report of a patient presented with unilateral raccoon eye, a quiet rare presentation, diagnosed with MM.

## Case Report

**Figure 1:**
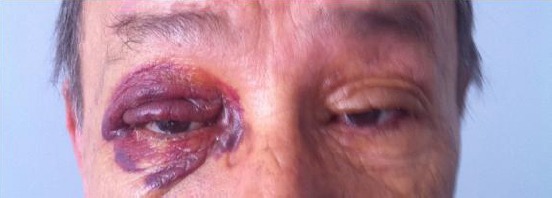
*Unilateral raccoon eye at presentation*.

A 64 years old male patient presented with an ecchymotic lesion in the right orbita and bilateral proptosis about 1 week to internal medicine outpatient (Figure 1). He has a weakness about 5 months. He denied any history of trauma, vision disorders, and/or anticoagulation treatment. There were no history of chronic diseases such as diabetes mellitus, hypertension, coronary artery disease and history of surgery, and bleeding disorders.

On the physical examination, he was afebrile, and vital signs were stable. Tongue of the patient was in normal size. Electrocardiogram did not reveled any arrhythmia, and loss of voltage. Patient did not define any neuropathy. There was an ecchymotic lession about 3-4 cm. diameter in the right orbita without any ecchymotic lesions (petechie, purpura, or any type or bruise) in the rest of the body. Peripheral lymphadenopathy was not observed; liver and spleen were not palpable. Hematologic parameters were found normally; Hemoglobin 12.9 gr/dl, hematocrit 36.7%, platelets 377,000/mm^3^, white blood cell 8,730/mm^3^, Biochemical parameters such as urea, creatinine, serum electrolytes, AST, ALT, uric acid, TSH, fT3, fT4 levels were found in the normal range. Total protein was 6.5 gr/dl (normal range: 6.4-8.3 gr/dl), albumin was 2.4 gr/dl (normal range: 3.5-5 gr/dl), globulin was 4.1 gr/dl. ESR level was found 92 /h. The patient was referred to the hematology outpatient for further investigation. Gamma Globulin 36.2 % (normal range: 10.7-20.3), Beta globülin 7.5 % (normal range: 8.6-15.6), alfa-1 globulin 2% (normal range: 1-3), alfa-2 globulin 12.3% (normal range: 9.5-14.4), albumin 42% (normal range: 52-65), total protein 7.3 gr/dl (normal range: 6.3-8.7). Albumin/globulin ratio was 0.72. Serum IgG level was 33.7 gr/L (normal range: 7-16), IgM level was 0.639 gr/L (normal range: 0.4-2.3), IgA level was 0.606 gr/L (normal range: 0.6-3.09 g/L), Lambda level was <0.05 g/L, and Kappa level was 1.8 g/L (normal range: <0.03 G/L) in the serum. Electrophoresis of serum and concentrated urine were performed. In Urine immunofixation electrophoresis urine κ concentration was found 0.9 mg/dl (normal range: < 1.5 mg/dl), urine λ concentration was found 0.3 mg/dl (normal range: < 1.5 mg/dl), and κ/λ ratio was found 3 (normal range: 0.75-4.5). PTT was 15.7 sec. aPTT was 37 sec.

**Figure 2:**
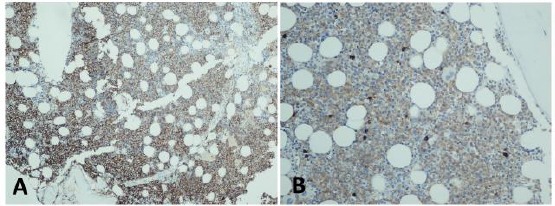
*Bone Marrow Biopsy. A. Atypical diffuse plasma cell infiltration with CD138 positivity. B. Atypical diffuse plasma cell infiltration with Kappa positivity*.

Fibrinogen level was 354 mg/dL. Echocardiography demonstrated 10 mm of end diastolic interventricular septal thickness. Serum Tropoinin T level was in normal range. BNP was <10 pg/mL. The bone marrow aspiration&biopsy was performed. Histopathological examination demonstrated 30% of plasma cell accumulation in bone marrow with diffuse positive staining with CD38 and kappa (Figure 2A and 2B).

Congo Red staining of bone marrow did not reveled any sign of amyloidosis. PET-CT did not demonstrate any mass resembling. Plasmocytoma. diagnosis of IgG kappa multiple myeloma was established. Two doses of combination of Vincristine, Doxorubicin (Adriamycin) and Dexamethasone (VAD), then four doses of combination of bortezomib, cyclophosphamide and dexamethasone (VCD) were used in the treatment. Patient was in complete remission after the treatment. Autologous stem cell transplantation was planned for the patient.

## Discussion

Multiple myeloma (MM) comprises 1% of all malignancies and 10% of hematologic malignancies [6]. The incidence increases with age and reaches a peak in the 60-70 years of age [7]. The incidence of MM is approximately 50 per million and slightly more common in men [8]. Our patient was a 64 year old man.

The most common presenting symptom of MM is bone pain due to lytic bone lesions.

AL type amyloidosis is a monoclonal disorder with an incidence 10/1×10^6^ [9]. AL type amyloidosis occur in 10-15% of plasma cell disorders [9]. AL amyloidosis could be as a result of monoclonal plasma cell disorder or as well as without any proved elevated monoclonal plasma cells. Paraprotein tissue depositions could be observed in diffuse or localized forms. Cardiac, renal, hepatic, peripheral nervous system, and soft tissue involvement could be observed in AL amyloidosis [10]. Light chain (AL) type amyloidosis is one of the reasons that can result raccoon eyes, because of either soft tissue involvement or factor deficeiency [10]. The prevalence of AL amyloidosis is 5-10 folds lower than multiple myeloma.

The classical triad of raccoon eyes composed of unilateral/bilateral rapidly progressive proptosis, periorbital ecchymosis and edema. This presentation could be seen in the skull base trauma, orbital metastasis, myxedema, amyloidosis, multiple myeloma, lymphoma or hemophilia. Our patient did not have a history of trauma, or thyroid diseases.

Our patient was presented with unilateral raccoon eye, a quiet rare presentation. Laboratory findings revealed high ESR levels. This finding was consistent with MM. Other findings such as anemia, acute renal failure were not found in our patient. Because amyloidosis is one of the etiological factors for raccoon eye, we excluded the probability of amyloidosis with absence of typical physical examination findings such as orthostatic hypotension, and macroglossia; Congo Red staining of bone marrow, and echocardiographic imaging.

In conclusion, Raccoon eye is a quiet rare presentation of patients with MM. Physicians should suspect the diagnosis of MM, in the case of raccoon eye even unilateral presentation without a history of orbital or skull trauma in elderly patients.
